# Editorial Correspondence

**Published:** 1879-08

**Authors:** 


### EDITORIAL CORRESPONDENCE.

New Orleans, La , July 29th, 1879.

*Dr. J. G. Westmoreland, Atlanta, Ga:*

*My Dear Doctor:* When I left Atlanta for Memphis last January, I promised you to examine into the sanitary conditions of the latter city, and give you the results of my investigations and impressions, especially with reference to the bearing these conditions may have upon the origin and spread of yellow fever.

This promise has awaited its fulfilment until now, and though the facts I have gathered are not sufficient to justify very definite conclusions, I will attempt to convey to you a correct notion of both.

The city of Memphis is built upon a ridge, known as the Chickasaw bluffs, running parallel with the east bank of the Mississippi river. The bluff at the river front is from fifty to seventy-five feet above average high-water. From here it slopes eastwardly to a natural drain, known as Bayon Gayoso. From the Bayon the rise is again gradual to beyond the city limits.

Bayon Gayoso which is the common carrier of all the filth which can conveniently be conducted into it, flows sluggishly northward, and empties into the Wolf river, a small tributary of the Mississippi. All this filth then flows past the city front on its way down the river, and, during westerly winds, its mephitic gasses and odors are wafted over the city a second time. The remedy for these evils would be to cut a canal from the head of the bayou to the Mississippi which could be done at comparatively little expense. A constant current of fresh water from Wolf, which could easily be diverted from its natural outlet, would then sweep all the filth out of the bayou into the Mississippi; the waters of this river would swell the current of the Wolf, and give the bayou a thorough flushing. As the high water generally occurs in the spring or early summer, these flushings would come just at the time when most needed, and leave the drainage canal pure and clean during the dangerous hot season.

The privy system, that abomination of sanitarians everywhere, is the only one in use in Memphis for the disposal of human excrement. Frequently the vaults are not emptied until they are full to overflowing, when a heavy rain frequently washes the filth out and spreads it over the ground round about. As the cisterns from which most of the inhabitants obtain their drinking water are underground, and frequently in close proximity to these filth receptacles, it may be easily imagined that the water used by the Memphis people is none of the purest.

On the opposite, western side of the river from Memphis, the Arkansas lowlands and swamps extend back for many miles. At every considerable flood in the Mississippi river, these lowlands are covered by the water, which deposits a large quantity of the alluvion brought down from above. When the water subsides, and the summer heat begins to exert its effects upon the decomposing vegetable matter contained in this sediment, malaria is generated and carried over the city by the westerly winds that prevail during the latter part of summer. If you object to the term malaria as an unknown quantity, I will say, those local conditions of the atmosphere are produced which promote the development of malarial fever.

This summary of the unsanitary condition of the sadly stricken city may furnish a basis for certain conclusions respecting the causation of yellow fever.

In the first place, the condition of Memphis in 1878, and in a less degree, during the present year, was strikingly similar to that of Savannah in 1876, so truthfully and graphically described by surgeon Alfred A. Woodhull, United States Army, in his report upon the epidemic of yellow fever in that city, published in the *American Journal of Medical Science*, July, 1877.

Second, the epidmics of 1876 and 1878 probably, and the one at Memphis this year, certainly, prove that yellow fever when occurring in this country, is not always imported, but may be of local origin. (Several cases have within the last two days occurred in this city—two fatal. No connection can be traced to an exotic origin.)

I am not ready to admit, however, that yellow fever is merely a variety of malarial fever, or that it is produced by the same causes that promote the development of our common paludal fevers. Its mode of propagation would alone, it seems to me, destroy this theory.

Thus, in New Orleans, several epidemics have occurred in which the fever was confined to certain districts, in some cases even being limited to one side of a street. If the cause of yellow fever (call it a "germ" if you will) were volatile and transportable in and by the atmosphere, such strict limitations could not occur. In ordinary malarial fevers we know in fact that they do not occur. The cause of the spread—if not of the origin—of yellow fever must therefore, I feel assured, be sought in what Col. Woodhull, in the report above referred to, terms "air and pollution;" that is, air saturated with the gaseous products of the decomposition of animal, and perhaps vegetable, matter. It is true these conditions are not alone sufficient to propagate the disease. Certain meteorological elements, as long-continued high temperature and probably a high humidity, are also necessary, but the latter conditions cannot be obviated while the former may.

If these facts were more clearly apprehended and considered by our health authorities, and more attention paid to the ventillation of houses, the cleaning of streets, disposal of sewage and the furnishing of an adequate supply of pure water, I believe we would not only soon see the last of those senseless quarantines which so disgrace sanitary science, destroy the commerce and prosperity of communities and outrage humanity, but that we could confidently look forward to the gradual extinction of epidemics of all kinds. Very truly yours,

George H. Rohe.

Our esteemed correspondent has in the above communication presented some important facts connected with the origin of yellow fever in Memphis. What produced it in Memphis does most certainly produce it everywhere.

Dr. Rohe from such facts has formed just conclusions, but has in some way got the idea of propagation, which the facts certainly do not warrant. He gives the fact of one side of a street being subject to the disease while the other is not, as evidence of spreading of the disease by propagation. When the behavior of malaria is known the explanation is easy. Malarial poison is wafted by a breeze and is arrested by obstructions, such as walls, thick foliage, etc. One side of a street may receive the breeze while the other does not, and thus have the disease-producing poison inhaled by the people on that side. How can this fact be explained on the germ propagation theory? Does the germ pass along brick walls and pavements and not through McAdamized or dirt streets? If from one person to another, do not the people of opposite sides communicate with each other?—[Ed.

---

Big Cane, St. Laudry Parish, La., June 15, 1879.

*Dr. J. G. Westmoreland, Atlanta:*

Dear Sir—Allow me to add my mite as an M.D. to all who may have an opportunity of trying or testing its merits. I have for several years, in most of the cases I here note, used concentrated ammonia and chloroform, equal portions, as an application for bites of poisonous reptiles, sting and bite of poisonous insects, with perfect success; and last, but not least, I used the same preparation in many cases of individuals bitten by rabid animals, mad dogs, with perfect success, and have to see the first case that showed even symptoms of hydrophobia. I am satisfied the ammonia of itself is sufficient to destroy the virus by neutralizing every particle, so injected into the flesh. Sir you are aware that the treatment laid down in our text books, is to extirpate or cut away the parts if possible. Should you see proper in your judgment to test the remedy or publish it that others may try it, you will certainly find that it will do all that I claim for it. I have many cases of note in my book, one in particular. The first a dog owned by George Fleshman, a near neighbor, was bitten by another rabid dog, which I shot and killed on my front veranda, a few moments afterwards. The dog is yet alive, and has never shown any symptoms of hydrophobia; also a negro woman in the neighborhood, I have treated over two years since.

Lest I tax or weary you, I will close, by saying please notice this to your satisfaction. I claim that the ammonia neutralizes the virus, and chloroform added acts medically for causing a flow of blood, if the surface be much abased.

Yours, respectfully,

W. B. REYNOLDS.

